# Exploring undergraduate medical students’ perception of an integrated longitudinal research curriculum within a competency-based framework

**DOI:** 10.1371/journal.pone.0343409

**Published:** 2026-02-23

**Authors:** Farah Ennab, Reem Hatem Abdulkareem, Rachid Kaddoura, Ayatullah Hegazy, Mouza Lootah, Yajnavalka Banerjee, Stefan S. Du Plessis, Tom Loney, Amar Hassan Khamis, Aida Joseph Azar

**Affiliations:** 1 Institute of Learning, Mohammed Bin Rashid University of Medicine and Health Sciences, Dubai Health, Dubai, United Arab Emirates; 2 College of Medicine, Mohammed Bin Rashid University of Medicine and Health Sciences, Dubai Health, Dubai, United Arab Emirates; 3 Hamdan Bin Mohammed College of Dental Medicine, Mohammed Bin Rashid University of Medicine and Health Sciences, Dubai Health, Dubai, United Arab Emirates; Alexandria University Faculty of Nursing, EGYPT

## Abstract

**Background:**

Embedding research training in undergraduate medical education strengthens analytical skills, scientific reasoning, and evidence-based practice, yet such training is often short-term or elective, with limited evidence from the Middle East. At our medical university, a mandatory longitudinal research curriculum was implemented with progressive skill development. Building on our previous work on the sequential integration of research methods, this study evaluates students’ perceptions of the program.

**Aim:**

To investigate the perceived value, impact, and barriers of a longitudinal research curriculum among undergraduate medical students.

**Methods:**

A cross-sectional survey was conducted among students who completed five compulsory research courses. The curriculum provided structured competencies through integrated content, protected time, and formal supervision. Data were collected using a self-administered questionnaire comprising demographics and 31 items rated on a 7-point Likert scale, assessing research perceptions and scholarly activities. Exploratory factor analysis identified five domains. Internal consistency (Cronbach’s alpha) and sampling adequacy (KMO test) were confirmed, with factor loadings ≥0.50 considered acceptable (p < 0.05).

**Results:**

A total of 106 students completed the survey (67.9% female, 32.1% male; mean age 23 years (SD = 1.8)). Overall perceptions toward research were positive, total mean score of 162.17 (SD = 17.12), corresponding to 74.5% agreement. The highest-rated domains were positive attitude toward research (mean = 37.62, 89.6%), perceived relevance (mean = 40.30, 85.7%), and perceived necessity (mean = 37.14, 75.8%), indicating strong endorsement of research for clinical training and career development. Challenge-related domains showed moderate agreement (61.2%), reflecting research barriers and dissemination challenges.

**Conclusion:**

Students strongly endorsed structured research training for professional competency development, and despite reported challenges, demonstrated a positive research orientation suggestive of sustained engagement. This study provides a novel evaluation of a mandatory longitudinal research curriculum in the Middle East. It addresses a critical regional gap and offers a model for integrating research training into undergraduate medical programs that support the development of clinician–scientists.

## 1. Introduction

Research education is widely recognized as a fundamental component of undergraduate training across disciplines, including medicine, engineering, and the social sciences, where it fosters creativity, critical thinking, and evidence-based practices [1–4]. In medical education specifically, structured research experiences play a pivotal role in cultivating analytical skills, scientific reasoning, and habits of lifelong learning, ultimately preparing future physicians to contribute to medical advancement and improve patient care [5,6].

Research training at the undergraduate level enriches educational experiences, fosters evidence-based clinical competencies, and is vital for developing clinician-scientists capable of advancing both patient care and medical innovation [1,7]. Early and longitudinal integration of research education within undergraduate medical curricula has been associated with a higher likelihood of continued scholarly activity after graduation [8,9]. It also enhances academic productivity, bridges the gap between theoretical knowledge and clinical application, and strengthens students’ foundation for academic and professional success [10]. Research participation further fosters critical thinking, research skills, and the development of a mindset that extends into professional practice [11,12].

The positive impact of research engagement is reflected across multiple domains. For patients, clinician involvement in research improves the quality of care by facilitating the application of evidence-based findings into practice [7]. For medical trainees, research experience sharpens critical thinking, strengthens clinical reasoning, and broadens professional opportunities [13]. Active participation in research has also been shown to significantly improve students’ appreciation for research education, emphasizing the importance of experiential learning in shifting perceptions and building research confidence [14–16]. Moreover, engagement in research activities cultivates transferable skills such as communication, teamwork, and resilience, which are essential for clinical practice and leadership in healthcare settings [17]. In response to these recognized benefits, many medical schools globally have introduced structured research curricula to ensure that students acquire essential research competencies during their undergraduate training [10]. Similarly, in the Middle East and North Africa (MENA) region, efforts to integrate research training into medical curricula are increasingly visible [18,19]. However, challenges such as limited mentorship and insufficient institutional support continue to constrain student engagement in research activities [7,20–22].

Despite these advances, important gaps remain in understanding how longitudinal research training influences students’ engagement, attitudes, and perceived professional readiness beyond isolated research experiences [23]. Understanding students’ experiences and perceptions is essential for informing curriculum design and optimizing research training effectiveness [24]. Furthermore, while structured research curricula aim to foster competence and engagement, students may continue to encounter practical challenges in conducting research [25]. Understanding these barriers is essential for promoting resilient research engagement and tailoring educational strategies effectively [26,27]. Recent studies have shown that active participation in research significantly improves students’ appreciation for research education [28,29], highlighting the critical role of experiential learning in shifting perceptions, enhancing research confidence, and strengthening long-term engagement with research activities [30]. These findings underscore the need to investigate how experiential, longitudinal research programs impact medical students’ research attitudes and competencies. To address this gap, the present study aims to explore the perceived impact and barriers of a longitudinally embedded research curriculum on undergraduate medical students’ attitudes, orientation, and engagement with research at Mohammed Bin Rashid University of Medicine and Health Sciences (MBRU) in Dubai Health, Dubai, United Arab Emirates (UAE). It also examines how structured, early exposure to research supports the development of evidence-based competencies and informs the integration of research within undergraduate medical curricula.

## 2. Methodology

### 2.1. Educational setting and curriculum overview

This cross-sectional study was conducted at the College of Medicine (CoM), MBRU, an undergraduate medical university established in 2016. The first Cohort enrolled in the same year and graduated in June 2022. The CoM offers a six-year undergraduate Doctor of Medicine (MD) program comprising 12 semesters and structured into three distinct phases (Table 1).

**Table 1:** Structure and sequencing of the integrated longitudinal research curriculum in the undergraduate MD program at MBRU (Cohort 2022–2026).

Phase	Year	Semester	Course	Credit	Level of complexity	Duration (weeks)	Research Curriculum	Assessment	Course Delivery Year (Cohort 2022–2026)
Phase 1 *Normal structure function and behaviour*	Year 1	Sem 1	FEB1	1	Foundational	20	Theoretical foundations of research methodology and biostatistics	Formative: Written assignments Summative: Open book midterm and end-of-term examination	2016–2020
		Sem 2	FEB2	1	Foundational	20	Continuation of foundational concepts using active learning strategies	Formative: Written assignments Summative: Open book midterm and end-of-term examination	2017- 2021
Phase 2 *Abnormal structure function and behaviour*	Year 2	Sem 3	RM1	2	Reinforcement	20	Intermediate research training: students select projects from a vetted list	Formative: Written assignments Summative: Open book midterm and end-of-term examination	2017- 2021
		Sem 4	RM2	2	Independent research	20	Students begin independent research under faculty supervision	Formative: Written assignments Summative: Midterm examination; research proposal; oral presentation; supervisor performance evaluation (proposal and oral presentation double-graded using predefined rubrics)	2018- 2022
	Year 3	Sem 5	RP	3	Independent research	20	Completion and presentation of research	Formative: Written assignments Summative: Research dissertation; poster presentation; supervisor performance evaluation (dissertation and poster presentation double-graded using predefined rubrics)	2018- 2022
		Sem 6	–	–	Transitional phase	20	Research consolidation and clinical readiness	–	2019-2023
Phase 3 *Clinical practice*	Years 4–6	Sem 7–12	–	–	Application in clinical setting	120	Integration of research in clinical practice	–	2019-2026

Note. Sem, semester; Grad, graduation; FEB, Fundamentals of Epidemiology and Biostatistics (Courses 1 and 2), delivered in Year 1 (Semesters 1 and 2); RM, Research Methods (Courses 1 and 2), delivered in Year 2 (Semesters 3 and 4); RP, Research Project (Course 5), delivered in Year 3 (Semester 5).

This table presents the structured progression of the medical curriculum, emphasizing the integration of research components across various phases. Spanning Phases 1 and 2, five integrated research courses cumulatively account for 9 credit hours, guiding students from foundational research principles to independent project execution. The inaugural cohort commenced studies in August 2016, culminating in graduation by June 2022. Subsequent cohorts have followed this annual progression, ensuring a consistent integration of research education throughout the program.

The initial two phases of the program, Phase 1 (Year 1) and Phase 2 (Years 2 and 3), focused on cultivating a robust grounding in biomedical sciences. Coursework encompassed key domains in medicine such as biochemistry, anatomy, pathology, pharmacology, microbiology, research methodology, and biostatistics, alongside other core pre-clinical subjects [31,32]. The pre-clinical courses were thoughtfully structured to scaffold the cognitive and analytical skills for transition into Phase 3 (Years 4–6), which centered on hands-on clinical clerkship training across a diverse healthcare landscape in the UAE. The MD curriculum was designed using spiral framework, allowing iterative exposure to key concepts at increasing degrees of complexity, thereby deepening the student’s understanding and promoting knowledge and skills acquisition across the continuum of the program [33].

### 2.2. Structure, phases, and assessment of the integrated research curriculum

In the first two phases of the MD program, MBRU established research as a core pillar of medical education by embedding a longitudinal research curriculum consisting of five courses delivered over the first five consecutive semesters of the preclinical years (Years 1–3), totaling 9 credit hours. These included Fundamentals of Epidemiology and Biostatistics (FEB) 1 and 2, Research Methods (RM) 1 and 2, and Research Project (RP) (Table 1) [30]. The foundational phase of this curriculum began in the first semester of Year 1 with courses FEB1 and FEB2. At this stage, students typically had no prior exposure to research. This phase was to introduce students to the basic principles of research methodology and biostatistics, delivered through brief lectures and reinforced through case-based learning [34], small group activities [35], and active learning strategies [36].

In Phase 2, during Semester 3 (course RM1), students began intermediate research training by selecting a research project from a curated list of faculty-proposed topics that included a short description of the project and corresponding supervisor. All topics were pre-reviewed by a dedicated committee to ensure feasibility within the curriculum timeline and alignment with academic standards. Students met with the potential supervisors to discuss the scope of the projects before selecting their research topic and supervisor. In Semester 4 (course RM2), students transition to applied, student-led research under the mentorship of MBRU faculty and affiliated faculty across academic, clinical, and allied health disciplines (Table 1). These projects span diverse areas, including laboratory research, clinical studies, and socio-behavioral research in health professions education, with a focus on project planning, data collection, and critical thinking skills as they engage in independent research work.

In Phase 2, during Semester 5 (course RP), the final course of the research curriculum, students consolidated their research experience and brought their project to completion. This stage marked the culmination of the five integrated research courses embedded within the broader programmatic assessment framework at MBRU (Table 1) [37]. Students were required to complete a dissertation in the format of a peer-reviewed scientific manuscript and present their findings as conference-style posters. Across the five courses, students engaged in all stages of the research process including literature review, formulation of research questions, study protocol development, Institutional Review Board (IRB) approval, data collection and analysis, and scientific manuscript preparation. These activities were designed to support scholarly identity and enhance research literacy among graduating medical students [38].

Student assessment across the five integrated research courses was aligned with course learning objectives and comprised both formative and summative components (Table 1). Formative assessment in all semesters included written assignments, while summative assessments contributed to students’ grade point average (GPA). Summative assessment was continuous across the curriculum. In the first three courses (FEB1, FEB2, RM1), summative assessment consisted of an open-book midterm examination and an end-of-term examination (Table 1). In Semester 4 (course RM2), summative assessment included a midterm examination, submission of a research proposal, an oral presentation, and supervisor evaluation of student performance. The research proposal and oral presentation were double-graded by independent faculty members using predefined rubrics. In Semester 5 (course RP), assessment included supervisor evaluation of students’ performance, evaluation of the research dissertation, and a poster presentation delivered in a university conference setting; the research dissertation and poster presentation were also double-graded using standardized rubrics by two independent faculty members (Table 1). This approach ensured objectivity, consistency, and reliability.

During the clinical years (Years 4–6), students were expected to extend and apply the research competencies acquired during the preclinical phase through practical activities such as critical appraisal of literature, conducting clinical audits, and participating in scholarly projects (Table 1). This alignment between preclinical and clinical phases ensured continuity in skill development and facilitated the application of evidence-based principles in real-world clinical settings. Students built on their earlier training by engaging in formulating research questions, selecting suitable methodologies, and contributing to quality improvement efforts [39].

### 2.3. Research curriculum design and educational theories

Instead of being offered as a standalone track, this research curriculum was embedded within the broader MD program, ensuring that undergraduate medical students were introduced to research principles early in their medical education. At the start of each semester (Semesters 3, 4, and 5), students and supervisors received structured supervision guidelines, including briefings, templates, and a timeline outlining SRP objectives, key deliverables, and the roles and responsibilities of both students and supervisors. To further support students, a weekly student research clinic provided individualized guidance throughout the academic year.

An introductory session at the beginning of Semester 3 oriented students to the structure, expectations, and assessment procedures of the SRP, which was delivered through three longitudinal research courses (RM1, RM2, RP) (Table 2). The research curriculum, developed using a design-based research approach [40], was grounded in Kolb’s Experiential Learning Theory [41] and the principles of social constructionism [42]. These frameworks emphasize experiential, reflective, and interactive learning and informed the design through iterative, practice-based opportunities aimed at fostering critical thinking, reflective practice, and applied research skills. The frameworks were operationalized through guided research tasks embedded across the curriculum. For example, students applied taught epidemiology, biostatistics and research concepts by formulating research questions, critically appraising the literature, and collecting and analysing data using SPSS statistical software package. This was followed by structured feedback and collaborative discussions with peers and faculty.

**Table 2:** Roles and responsibilities in the integration of the student research project across the MBRU-MD curriculum (Semesters 1–5).

Semester	Educational Focus	Students Role	Supervisors Role
Sem 1, 2	Introduction to research methodology, epidemiological principles, and foundational biostatistics with early exposure to SPSS for data analysis. Emphasis is placed on developing critical thinking skills through inquiry-based learning.	Attend weekly FEB sessions. Engage in foundational learning through lectures, inquiry-based and active learning strategies. Participate in group-based activities and individual assignments. Develop skills in epidemiology, biostatistics, SPSS, and critical thinking.	N/A
Sem 3	Understanding project structure and aligning research topics with student interests. Students engage in more advanced instruction in SPSS and research methodology to prepare for independent project work in the following semester.	Attend SRP orientation and weekly RM1 sessions. Explore approved research topics, rank preferences, confirm allocated project and supervisor, and begin background reading.	Attend SRP orientation. Submit research topics to SRWG. Confirm supervision and provide early-stage mentorship in accordance with the SRP framework.
Sem 4	Application of ethical research principles through IRB submission and structured proposal development. Students begin data collection and build competencies in research ethics, protocol adherence, project planning, and scholarly communication.	Attend weekly RM2 classes and utilize SR-C for ongoing support. Complete mandatory ethics training. Draft and finalize the research proposal. Submit the IRB application via Cayuse portal and begin data collection once approved. Deliver an oral presentation of research progress and meet regularly with the supervisor.	Guide students in drafting research proposals and preparing IRB applications. Ensure adherence to ethical standards and provide regular feedback. Oversee the initiation of data collection and assess student progress.
Sem 5	Develop analytical, interpretation, and scientific communication skills.	Attend weekly RP classes and access SR-C for support as needed. Finalize data collection and conduct analysis with supervisory input. Draft, revise, and submit a manuscript-format dissertation and poster. Present research at the Annual College of Medicine Student Research Day. Pursue optional publication or conference submission beyond course requirements.	Guide students through final stages of data collection and initial analysis in line with SRP guidelines. Support development of discussion and interpretation of results. Provide feedback on manuscript drafts and assess overall project quality. Attend Student Research Day presentations and assist with publication efforts.

Note.Sem, semester; FEB, Fundamentals of Epidemiology and Biostatistics; SRP, Student Research Project; RM, Research Methods (Courses 1 and 2), delivered in Year 2 (Semesters 3 and 4); RP, Research Project (Course 5), delivered in Year 3 (Semester 5); IRB, Institutional Review Board; SRWG, Student Research Working Group; SR-C, Student Research Clinic, is a supportive service available weekly during the academic semester, where students can receive guidance on any aspect of their research.

This table outlines the evolving roles of students and supervisors involved in the Student Research Project (SRP) from Semesters 1–5. The SRP is implemented through five integrated research courses that constitute a longitudinal component of the MD curriculum. These courses provide structured, weekly instruction in research methods and project development, culminating in the presentation and, where appropriate, dissemination of findings. The table serves as a practical framework to align expectations and support effective supervision throughout the research training continuum.

Research competencies were progressively scaffolded across the five research courses to support the development of transferable skills essential for evidence-based medicine and scholarly inquiry [43]. While publication in peer-reviewed journals was encouraged, it was not required for course completion, allowing students to focus on developing foundational research skills.

### 2.4. Study design

This study represents the second part of a larger research project evaluating the longitudinal research curriculum at MBRU. The first part, reported by Otaki et al (2023) [30], used a convergent mixed-methods design to examine undergraduate medical students’ journey through the mandatory five-integrated research module. Thematic analysis generated four sequential themes describing how students integrated the scientific research method: (1) Attend–Acquire, (2) Accumulate–Assimilate, (3) Apply–Appreciate, and (4) Articulate–Affect. Quantitative analysis identified two distinct GPA clusters (p < 0.01). In addition, joint display analysis integrating qualitative and quantitative findings informed the development of the 8A-Model [30]. Part 1 study highlighted the importance of embedding experiential research modules in medical curricula. Building on this foundation, the current study (Part 2) conducted a psychometric evaluation of students’ perceptions using exploratory factor analysis. Part 3, currently in progress, will examine students’ research productivity and identify potential enablers and barriers to sustained scholarly activity.

### 2.5. Eligibility criteria

Eligible participants were undergraduate medical students enrolled in the MD program at MBRU who had successfully completed all five integrated, longitudinal research courses delivered during the pre-clinical phase of the curriculum (Table 1). At the time of data collection, this included students from Cohorts 2022–2026. Students who had not completed all five integrated research courses at the time of data collection were excluded.

### 2.6. Survey instrument design, administration, and data collection

A criterion-based sampling strategy [44] was employed to recruit medical students who had completed all five research modules. Eligible students were invited to participate via their institutional MBRU email accounts, and the survey link was distributed electronically using Microsoft Forms. Prior to completing the survey, participants received an information sheet outlining the study’s purpose, its voluntary nature, and a maximum completion time of ten minutes. Participant recruitment took place from 01/09/2023, following institutional review board approval and students’ return from summer break, to 30/06/2024, during which the survey was sent to all eligible students and periodic reminders were issued to maximise response rates. The survey was self-administered, a format that reduces the potential for interviewer or facilitator bias and helps ensure more objective responses [45]. No identifiable personal data was collected, ensuring participant anonymity. This observational study was conducted in accordance with the ethical standards outlined in the Declaration of Helsinki [46].

A survey instrument consisting of 45 items was developed for this study. It was designed to assess students’ perceptions, attitudes, and engagement with research, including perceived barriers and the impact of structured, longitudinal research training on the development of evidence-based competencies. The instrument included demographic variables such as age, gender, country of citizenship, and graduating cohort, as well as open-ended (free-text) questions. The core of the instrument comprised 31 items measured using a 7-point Likert-type scale (1 = Strongly Disagree to 7 = Strongly Agree). These Likert-scale items were designed to capture students’ perceptions of research within a competency-based framework, along with their experiences related to research projects, publications, and scholarly outputs. Importantly, our study did not use a completely self-designed questionnaire, rather, it was adapted from a previously validated Likert-scale instrument [47] to ensure alignment with the specific context and objectives of our longitudinal research curriculum. To ensure appropriateness of our setting, content validity [48] was further established through independent expert review by three medical education faculty members, who provided structured feedback on clarity, relevance, and alignment with curricular goals. Based on their feedback, items were revised to improve comprehension, and construct consistency. The survey constructs were categorized into five domains: 1) Perceived relevance of research for professional development, exploring how students view research as a tool for career advancement; 2) Barriers to research confidence and resilience, identifying perceived obstacles such as time constraints or lack of mentorship; these reflect students’ subjective views rather than those of the institution; 3) Positive orientation toward research, assessing intrinsic motivators for research engagement; 4) Perceived necessity of research in academic and professional practice, examining the perceived value of research for medical training and career goals; and 5) Practical challenges in conducting and communicating research, addressing difficulties students face in disseminating scholarly work.

Data was collected over a six-month period from January 1 to June 30, 2024. At the time of data collection, two cohorts (2022 and 2023) had already graduated from the program and were considered recent alumni, while the remaining students were still enrolled in the clinical phase of the six-year undergraduate medical program. This meant that the lag time between research module completion and survey administration ranged from a minimum of approximately six months (Cohort 2026) to a maximum of about five years (Cohort 2022).

### 2.7. Data analysis

The MD program at MBRU has a diverse study population [49]. The survey was disseminated to the entire population (N = 229) across five cohorts. Respondents’ demographics were compared with institutional data to assess consistency with the broader population. To assess the adequacy of the sample, we referred to the meta-analysis by Wu et al. (2022) [50], which reported an average online survey response rate of 44%. Given our entire population of 229 students across five cohorts (2022–2026), an expected of 101 responses would be adequate. Reliability and internal consistency were assessed by calculating Cronbach’s alpha coefficients for the overall survey and each subscale, with values of ≥ 0.70 considered acceptable for reliability [51]. Sampling adequacy was further examined using the Kaiser-Meyer-Olkin (KMO) test [52], and data suitability was confirmed by Bartlett’s Test of Sphericity [53].

Descriptive statistics were used to summarize demographic data and Likert-scale responses, and an Exploratory Factor Analysis (EFA) with principal component extraction was conducted to identify underlying constructs within the survey. Factor loadings ≥ 0.50 were considered acceptable for retention [47]. All statistical analyses were conducted using the Statistical Package for the Social Sciences (SPSS), version 29.0 (IBM Corp., Armonk, NY, USA). Statistical significance was set at p < 0.05.

### 2.8. Ethical approval

The study was approved by the relevant MBRU Institutional Review Board (Reference #MBRU IRB-2023–54) and by the Dubai Scientific Research Ethics Committee (DSREC), Dubai Health Authority (DSREC-06/2024_17). Prior to commencement of the study, all participants provided fully informed written electronic consent, confirming their voluntary participation and right to withdraw at any time without providing a reason. No minors were included in this study.

## 3. Results

### 3.1. Survey response rate, validity, and reliability analysis

The survey was disseminated online to the entire population of 229 undergraduate medical students (Cohort 2022–2026), and 106 completed the survey, yielding a response rate of 46.3%. This aligns with the average online survey response rate of 44% reported by Wu et al. (2022) [50], supporting the adequacy of the sample for analysis. Construct validity of the 31-item Likert scale was supported by an overall Kaiser-Meyer-Olkin (KMO) measure of sampling adequacy (MSA) value of 0.853 (range 0.766 to 0.902), along with a highly significant Bartlett’s Test of Sphericity (P < 0.0001), indicating that the data were suitable for factor analysis (Table 3).

**Table 3:** Exploratory factor analysis, validity, and reliability metrics for survey constructs among undergraduate medical students who completed the integrated research curriculum (N = 106).

Factor name (cumulative variance load %)	Survey item	Exploratory factor analysis		KMO & Bartlett	Cronbach’s Alpha
**Factor loading**	**Item influence**
**1.** **Perceived relevance of research for professional development (62.345%)**	I feel that students should receive training and information on designing and conducting research	0.880	7	0.766^**^	0.791
	Research will contribute to the advancement of my professional clinical career	0.533			
	Research helps me understand how my MD courses relate to my clinical practice	0.602			
	Students should be informed about the clinical and translational significance of research	0.583			
	Research is essential for becoming a safe and competent clinician	0.635			
	The learning objectives covered in the research courses have helped me develop my written and communication skills	0.556			
	Research is essential to evolve into an evidence-based medical practitioner	0.576			
**2.** **Barriers to research confidence and resilience (51.903%)**	I feel anxious and scared when designing or conducting research. I become nervous and scared when I must conduct or practice research	0.640	5	0.797^**^	0.761
	Analyzing research data causes me stress and anxiety	0.607			
	Conducting research and publishing for career advancement are stressful	0.589			
	When designing or conducting a research study, do you worry about making mistakes that could affect the validity of your findings?	0.556			
	Research can have a negative impact on my wellbeing by creating stress and anxiety	0.504			
**3.** **Positive orientation toward research (67.789%)**	I am interested in conducting and developing my own research projects	0.718	6	0.834^**^	0.902
	Conducting research is beneficial for most medical students	0.741			
	The skills taught and acquired in the research courses will benefit my future clinical practice and career	0.612			
	I am interested in learning about the specific requirements to become a successful researcher	0.560			
	I will empower my clinical practice with research	0.769			
	I am willing to commit time to a research project including study design, data collection, analysis, and write-up	0.667			
**4.** **Perceived necessity of research in academic and professional practice (60.753%)**	I feel that conducting research is not necessary for obtaining academic or clinical recognition	0.633	7	0.784^**^	0.576
	Research is not essential for me to fulfil my responsibilities in my current professional career	0.539			
	I intend to apply principles of research outside of my professional practice	0.650			
	Research is not required outside of my professional practice	0.513			
	Research training is an essential part of my professional clinical training	0.613			
	Research is not a key component for me to perform my responsibilities in my current professional career	0.647			
	Research-driven thinking is important outside of my professional practice	0.604			
**5.** **Practical challenges in conducting and communicating research (55.954%)**	I did not have adequate training to initiate my research design	0.692	6	0.826^**^	0.842
	I face challenges when analyzing research data, especially when it comes to statistical analysis	0.603			
	I find it difficult to write a research manuscript	0.549			
	I have difficulty understanding research design concepts	0.635			
	Conducting research can be difficult most of the time	0.498			
	Research is a complex and complicated activity	0.380			
**Total Scale**			**31**	**0.853** ^**^	**0.801**

Note. KMO, Kaiser-Meyer-Olkin measure of sampling adequacy; Bartlett, Bartlett’s test of sphericity; EFA, Exploratory Factor Analysis; Cronbach’s Alpha, Internal consistency reliability.^**^p < 0.001 for all KMO/Bartlett tests.

This table presents the results of an exploratory factor analysis (EFA) conducted on the survey instrument. Each factor is labeled with its name and the cumulative variance it explains. For each survey item, the corresponding factor loading is provided, indicating the strength of its association with the underlying factor. Items with factor loadings ≥ 0.50 are considered to have a strong association with the factor. The number of items per factor, the Kaiser–Meyer–Olkin (KMO) measure of sampling adequacy, and Cronbach’s alpha coefficients are also reported to assess the suitability of the data for factor analysis and the internal consistency reliability of each subscale, respectively.

Reliability analysis showed good internal consistency for the overall instrument, with a Cronbach’s alpha coefficient (α) of 0.801 (range 0.576 to 0.902) (Table 3). At the factor level, internal consistency ranged from acceptable to excellent: ‘*Positive orientation toward research’* (α = 0.902), followed by ‘*Practical challenges in conducting and communicating research* (α = 0.842), ‘*Perceived relevance of research for professional development’* (α = 0.791), ‘*Barriers to research confidence and resilience’* (α = 0.761), and ‘*Perceived necessity of research in academic and professional practice’* (α = 0.576). These findings reinforce the robustness of the survey instrument in assessing students’ attitudes towards research.

### 3.2. Exploratory factor analysis

EFA revealed five distinct categories: 1) Perceived relevance of research for professional development; 2) Barriers to research confidence and resilience; 3) Positive orientation toward research; 4) Perceived necessity of research in academic and professional practice; and 5) Practical challenges in conducting and communicating research (Table 3). Most items show factor loadings above 0.50, indicating strong correlations with their respective factors. The factor *‘Positive orientation toward research’* accounted for the largest proportion of explained variance (67.8%), highlighting its central role in explaining students’ perceptions of research.

### 3.3. Demographic characteristics

In our study, 106 students responded to the survey (Table 4). At the time of the survey, the total number of enrolled Cohorts was five, with the Cohort of 2022 representing the first graduating Cohort and the Cohort of 2026 the most recent. The number of respondents per Cohort ranged from 12 (Cohort of 2023) to 33 (Cohort of 2026). Most respondents were female 72 (67.9%), with 34 (32.1%) males. The mean age was 23 years (SD = 1.8). In terms of nationality, 38 (35.8%) students were UAE nationals and 68 (64.2%) were non-UAE nationals. Overall, the sample reflected the MD program’s demographic composition in terms of gender, age, and nationality. Most respondents 102 (96.2%) were pursuing their first undergraduate medical degree at MBRU. In terms of productivity in research, 44 (41.5%) of students reported having completed and published at least one article, including 28 (26.4%) who have published two or more. Additionally, 68 (64.1%) reported having at least one article under revision or in preparation. All 106 students (100.0%) presented at least one research poster, including 66 (62.3%) who presented two or more. Also, 74 (69.8%) delivered at least one oral presentation since joining MBRU. Notably, one-third of students, 35 (33%), reported receiving at least one research award, reflecting engagement in competitive research activities. Additional analysis showed that the first graduating cohort (Cohort 2022) did contribute more to research productivity, due to the longer lag time between research module completion and survey administration.

**Table 4:** Demographic characteristics and self-reported research productivity of undergraduate medical students who completed the integrated research curriculum (N = 106).

Characteristics of the participants	No. (%)
***Gender***	
Male	34 (32.1)
Female	72 (67.9)
**Age, years (**Mean (SD))	23 (1.8)
***Nationality***	
UAE national	38 (35.8)
Non-UAE national	68 (64.2)
***Graduating Cohort***	
2022	22 (20.8)
2023	12 (11.3)
2024	21 (19.8)
2025	18 (17.0)
2026	33 (31.1)
***Awarded degrees from other Universities***	
No	102 (96.2)
Yes	4 (3.8)
***Articles published***	
None	62 (58.5)
Only one	16 (15.1)
At least two	28 (26.4)
***Articles under revision or preparation***	
None	38 (35.8)
Only one	28 (26.4)
At least 2	40 (37.7)
***Posters presented***	
Only one	40 (37.7)
At least 2	66 (62.3)
***Oral presentations delivered***	
None	32 (30.2)
Only one	47 (44.3)
At least 2	27 (25.5)
***Winning a research award***	
No	71 (67.0)
Yes	35 (33.0)

### 3.4. Students’ perception of research importance

Students’ perceptions across five categories were explored: perceived relevance, research barriers, positive orientation, perceived necessity, and dissemination challenges (Table 5).

**Table 5:** Undergraduate medical students’ perceptions and agreement levels on the importance of research after completing the integrated research curriculum (N = 106).

Survey items	Mean (SD)	% Mean	Categories
**Perceived relevance of research for professional development**	**40.30 (4.55)**	**85.7**	**Ag**
I feel that students should receive training and information on designing and conducting research	6.54 (1.02)	93.4	Ag – S. Ag
Research will contribute to the advancement of my professional clinical career	6.29 (1.27)	89.9	Ag – S. Ag
Research helps me understand how my MD courses relate to my clinical practice	5.86 (1.52)	83.7	SW. Ag – Ag
Students should be informed about the clinical and translational significance of research	6.55 (0.78)	93.6	Ag – S. Ag
Research is essential for becoming a safe and competent clinician	6.04 (1.30)	86.3	Ag
The learning objectives covered in the research courses have helped me develop my written and communication skills	5.89 (1.42)	84.1	SW. Ag – Ag
Research is essential to evolve into an evidence-based medical practitioner	6.37 (1.04)	91.0	Ag – S. Ag
**Barriers to research confidence and resilience**	**21.41 (6.46)**	**61.2**	**N – SW. Ag**
I feel anxious and scared when designing or conducting research. I become nervous and scared when I must conduct or practice research	3.58 (1.82)	51.1	SW. Dis – N
Analysing research data causes me stress and anxiety	4.38 (1.83)	62.6	N – SW. Ag
Conducting research and publishing for career advancement are stressful	4.81 (1.73)	68.7	N – SW. Ag
When designing or conducting a research study, do you worry about making mistakes that could affect the validity of your findings?	4.93 (1.77)	70.4	N – SW. Ag
Research can have a negative impact on my wellbeing by creating stress and anxiety	3.70 (1.88)	52.9	SW. Dis – N
**Positive Attitude Toward Research**	**37.62 (5.69)**	**89.6**	**Ag – S. Ag**
I am interested in conducting and developing my own research projects	6.42 (1.05)	91.7	Ag – S. Ag
Conducting research is beneficial for most medical students	6.33 (1.52)	90.4	Ag – S. Ag
The skills taught and acquired in the research courses will benefit my future clinical practice and career	6.24 (1.22)	89.1	Ag – S. Ag
I am interested in learning about the specific requirements to become a successful researcher	6.14 (1.25)	87.7	Ag – S. Ag
I will empower my clinical practice with research	6.15 (1.19)	87.9	Ag – S. Ag
I am willing to commit time to a research project including study design, data collection, analysis, and write-up	6.35 (1.06)	90.7	Ag – S. Ag
**Perceived necessity of research in academic and professional practice**	**37.14 (5.98)**	**75.8**	**SW. Ag – Ag**
I feel that conducting research is not necessary for obtaining academic or clinical recognition	5.68 (1.66)	81.1	SW. Ag – Ag
Research is not essential for me to fulfill my responsibilities in my current professional career	5.55 (1.85)	79.3	SW. Ag – Ag
I intend to apply principles of research outside of my professional practice	5.92 (1.35)	84.6	SW. Ag – Ag
Research is not required outside of my professional practice	5.07 (1.75)	72.4	SW. Ag
Research training is an essential part of my professional clinical training	6.20 (1.32)	88.6	Ag – S. Ag
Research is not a key component for me to perform my responsibilities in my current professional career	2.97 (1.77)	42.4	SW. Dis
Research-driven thinking is important outside of my professional practice	5.76 (1.45)	82.3	SW. Ag – Ag
**Practical challenges in conducting and communicating research**	**25.70 (7.6)**	**61.2**	**N – SW. Ag**
I did not have adequate training to initiate my research design	3.07 (1.9)	43.9	SW. Dis – Dis
I face challenges when analysing research data, especially when it comes to statistical analysis	4.97 (1.76)	71.0	SW. Ag
I find it difficult to write a research manuscript	3.92 (1.87)	56.0	SW. Dis – N
I have difficulty understanding research design concepts	3.20 (1.81)	45.7	SW. Dis – Dis
Conducting research can be difficult most of the time	5.0 (1.45)	71.4	SW. Ag
Research is a complex and complicated activity	5.54 (1.31)	79.1	SW. Ag – Ag
**Overall**	**162.17 (17.12)**	**74.5**	**SW. Ag – Ag**

Note. SD, Standard deviation; SW. Dis, somewhat disagree; N, Neutral; SW. Ag, somewhat agree, Ag, agree; S. Ag, strongly agree.

This table summarizes students’ agreement levels on various aspects of research within the medical curriculum. Mean scores (SD) and % means are presented for each survey item, categorized under key themes. Responses were measured on a 7-point Likert scale ranging from 1 (Strongly Disagree) to 7 (Strongly Agree).

#### 3.4.1 Perceived relevance of research for professional development.

The seven items in the perceived relevance for professional domain yielded an overall mean score of 40.30 (SD = 4.55), corresponding to 85.7% of the maximum score, which falls within the ‘Agree’ range in the Likert scale (Table 5). Notably, 4 out of the 7 items were rated between ‘Agree and ‘Strongly Agree’. The highest-rated item was *‘students should be informed about the clinical and translational significance of research’* with a mean (SD) of 6.55 (0.78), or 93.6%. This was followed by *‘students should receive training on designing and conducting research’* with a mean (SD) of 6.54 (1.02), or 93.4%. The item *‘research is essential for evolving into an evidence-based medical practitioner’* received a mean (SD) of 6.37 (1.04), or 91.0%, while *‘research will contribute to the advancement of my professional clinical career’* had a mean (SD) of 6.29 (1.27), or 89.9%. These data indicate students’ strong support for the role of research in their clinical and professional development, particularly in becoming competent, evidence-based practitioners.

#### 3.4.2 Barriers to research confidence and resilience.

Regarding barriers to research confidence and resilience, this domain yielded an overall mean score of 21.41 (SD = 6.46), or 61.2% of the maximum possible score. This falls within the ‘Neutral to Somewhat Agree’ range and indicates moderate concern regarding research-related stress and uncertainty (Table 5). Most items were rated between ‘Neutral to Somewhat Agree’ range in the Likert scale. The highest-rated item was *‘I worry about making mistakes that could affect the validity of my findings’,* with a mean (SD) of 4.93 (1.77), corresponding to 70.4% of the maximum score. This was followed by *‘conducting research and publishing for career advancement are stressful’* 4.81 (1.73), 68.7%, and *‘analysing research data causes me stress and anxiety’* 4.38 (1.83), 62.6%. In contrast, lower mean scores were observed for *‘research can have a negative impact on my wellbeing’* 3.70 (1.88), 52.9%, and *‘I feel anxious and scared when designing or conducting research’* 3.58 (1.82), 51.1%. Overall, these findings suggest that while students did not report high levels of research-related stress, the variability in responses indicates that some experience uncertainty or lack confidence.

#### 3.4.3 Positive orientation toward research.

Meanwhile, the six items in the positive orientation toward research domain yielded an overall mean score of 37.62 (SD = 5.69), corresponding to 89.6% of the maximum possible score, falling within the ‘Agree to Strongly Agree’ range on the Likert scale (Table 5). Each item reflected consistently high levels of agreement. The highest-rated statement was ‘*I am interested in conducting and developing my own research projects’*, with a mean score of 6.42 (SD=1.05), or 91.7%. This was closely followed by *’I am willing to commit time to a research project including study design, data collection, analysis, and write-up’,* received a mean score of 6.35 (SD = 1.06), or 90.7%. Overall, the data indicate strong student engagement and a strong interest in research, also a clear willingness to actively participate in the full spectrum of research activities.

#### 3.4.4 Perceived necessity of research in academic and professional practice.

Regarding the academic and professional relevance of research, this domain yielded an overall mean score of 37.14 (SD = 5.98), corresponding to 75.8% of the maximum possible score, which falls within the ‘Somewhat Agree to Agree’ range (Table 5). Five out of the seven items were rated within this range, with the highest-rated statement being ‘*Research training is an essential part of my professional clinical training’* with a mean (SD) of 6.20 (1.32) or 88.6%. In contrast, the item ‘*Research is not a key component for me to perform my responsibilities in my current professional career*’ received the lowest mean score, 2.97 (SD = 1.77) or 42.4%, falling within the ‘Somewhat Disagree’ range. These data indicate that most students view research as a relevant part of clinical education and professional development. However, the observed variability in responses suggests that a smaller proportion of students remain uncertain about its necessity for their future careers.

#### 3.4.5 Practical challenges in conducting and communicating research.

The final category assessed the practical challenges undergraduate medical students face in conducting and disseminating research. The six items in this domain yielded an overall mean score of 25.70 (SD = 7.6), corresponding to 61.2% of the maximum possible score, which falls within the ‘Neutral to Somewhat Agree’ range (Table 5). The highest-rated item was ‘*Research is a complex and complicated activity’* at 5.54 (SD = 1.31), or 79.1% of the maximum possible score, followed by *‘I face challenges when analyzing research data, especially statistical analysis’,* at 4.97 (SD = 1.76), or 71.0%. The lowest mean (SD) of 3.07 (1.90), or 43.9%, was observed for the item ‘*I did not have adequate training to initiate my research design’.* These findings suggest that while students experience some difficulties during the early stages of research, data analysis, particularly statistical interpretation, emerges as a more consistent challenge.

## 4. Discussion

### 4.1. Key findings

Our study explored undergraduate medical students’ perceptions of research following the completion of an integrated, mandatory longitudinal research curriculum at our medical university in Dubai, Dubai Health, UAE [30]. Overall, students reported a positive attitude towards the importance of conducting research and recognized its relevance to their professional and academic careers. However, they also highlighted several challenges, particularly in research dissemination and those related to resilience and balancing research output with academic coursework. This observation suggests that while our students value research, they may still face challenges integrating it alongside their academic responsibilities.

One of the most prominent themes observed in our findings relates to how students perceive the role of research and its relevance to their professional development. Specifically, students consistently emphasized the importance of structured research training and early exposure to learning the research process as part of their undergraduate medical training. They also perceived research as highly relevant to their future clinical practice and research careers. This aligns with prior findings suggesting that early research involvement among medical students significantly increases the likelihood of continued scientific productivity post-graduation and is strongly associated with a sustained interest in academic career pathways [6,9,54,55]. In addition, studies by Murdoch-Eaton et al. [56] and Burgoyne et al. [57] have also highlighted the importance of research skills in enhancing employability and career prospects. While long-term outcomes are yet to be established in our current cohort of medical students, their early recognition of the value of research practices suggests that our curriculum could serve as a precursor to sustained scholarly activity and ongoing professional development [19]. Beyond career advancement, students also acknowledged that research participation not only improved their clinical and research practice but also helped improve their communication and social skills, which are essential for practicing clinicians. Prior studies echo this, demonstrating that research engagement enhances transferable skills such as teamwork, time management, and critical thinking [57].

While students acknowledged the importance of research to advance their future careers, they did not always view their experience as entirely seamless and stress-free. Interestingly, feelings of research-related anxiety and pressure were reported. This could be attributed to the nature of frequent and often stressful academic obligations during medical students’ training [58]. The reported variability of responses in our study may relate to a lack of immediate visibility of impact; the interplay between recognizing research value and experiencing research-related stress suggests a need for better scaffolding and psychological support during the research process training [59]. Institutional strategies such as peer mentoring, dedicated faculty-led research support clinics, and a balanced approach that reduces emphasis on publication as an endpoint while maintaining scholarly motivation can help alleviate performance pressure. These strategies are already actively implemented and continuously strengthened at our institution.

In parallel with concerns about confidence in conducting research, a strong and positive orientation toward research was also observed, with students expressing a high degree of interest in initiating independent projects beyond the formal curriculum. This finding reflects intrinsic motivation [60] and is consistent with published data from Kaur et al. [61], suggesting that students tend to be satisfied following the completion of a mandatory research course, particularly those who manage to publish their paper in a peer-reviewed journal. This phenomenon aligns with the ‘motivation theory’ [62], which suggests that successful experiences fuel continued participation and enthusiasm. In the context of our study, this applies to research endeavors. Students also agreed that research is beneficial for most medical students. One of the main factors may be due to the increasing competitiveness of residency applications, as research experience is often considered an advantage [63]. Notably, a study that sought to investigate the potential factors behind why medical students choose to pursue research during their medical school training found that the primary motivation was the desire to enhance their residency prospects and chances of matching into competitive specialties [64]. More recently, an increasing number of medical residency program directors are affirming the importance of research literacy in future resident applicants, as it demonstrates commitment to academic excellence and is an indicator of a physician’s ability to initiate and complete an independent project, potentially towards the publication stage [7].

Beyond personal motivation and emotional experience, students also demonstrated a clear understanding of the perceived necessity of research in academic and professional practice. Their responses reflect the consistent view that research constitutes a vital element of clinical training and is integral to fulfilling professional and clinical responsibilities [6]. This perception may stem from their early exposure (as early as Year 1) to a curriculum that explicitly emphasizes the important role of physicians to remain up to date with evolving medical literature and incorporate new findings of evidence-based medicine into their clinical practice [65]. A structured research curriculum that integrates theoretical instruction with practical hands-on project experience provides students with a robust foundation in scientific inquiry and critical appraisal [66]. This approach underpins the design and delivery of our program. The findings of our study indicate that students recognize the advantages of early research engagement, particularly in cultivating the analytical and reflective skills necessary for becoming a well-rounded and competent physician [67]. The longitudinal research module at MBRU was strategically integrated into the preclinical phase of the MD curriculum to provide students with structured, early exposure to research principles and practices. Results from the exploratory factor analysis and perception data indicate that students highly valued this early engagement, with strong agreement on items related to the role of research in clinical reasoning, communication skills, and the development of evidence-based competencies. These findings reinforce the importance of embedding research training early in undergraduate medical education and offer empirical support for the continued integration of research-focused learning outcomes into the broader curriculum.

### 4.2. Curriculum improvement

While students valued formal training to engage in research projects, they also highlighted several practical challenges that may impede their research output, particularly with respect to dissemination and biostatistical literacy. Although many expressed confidence in writing manuscripts, a substantial number of students struggled in analyzing research data, especially when it came to statistical analysis. This is consistent with previous studies highlighting statistical analysis as one of the most difficult aspects of research for medical students [68]. Windish et al. [69] found that many medical students had limited confidence in interpreting statistical results, which hindered their ability to critically appraise the literature and conduct independent research. Similarly, Cam Ha et al. [70] reported that students who struggled with biostatistics were less likely to engage in research activities. This study also observed that students who reported a greater interest in learning more about biostatistics and an increased knowledge of manipulation of statistics were more likely to experience an increased inclination toward a research career. Due to the time constraints that medical students face [71], research curricula should place greater emphasis on statistical training. Consequently, a study by Siemens et al. [72] investigating students’ attitudes toward research, found that only 15% of students felt that there was sufficient training in research methodology and statistical analysis in medical school. Improving statistical literacy among medical students is, therefore, a critical component of fostering research engagement. Lastly, the process of conducting research and understanding the nuanced distinctions between various study designs remains a critical area of discussion in undergraduate medical education, particularly when navigating methodological frameworks, statistical interpretation, and ethical considerations [73]. To address this, medical institutions should actively work on incorporating strategies to mitigate the underlying complexity of research training and provide scaffolded and iterative training opportunities for medical students across the program.

Taken together, these findings suggest that strengthening undergraduate research curricula requires attention not only to formal research training, but also to how students are supported in developing confidence and competence in applying research skills, particularly in biostatistics and data interpretation. Embedding scaffolded and iterative opportunities for statistical learning, alongside structured guidance for research dissemination, may help align curricular expectations with students’ developmental needs. Such approaches may support sustained engagement in research while reducing barriers related to confidence, workload, and methodological complexity.

### 4.3. Strengths and limitations

A key strength of our study is its focus on a well-characterized sample of medical students across multiple cohorts (in preclinical and clinical phases), all of whom completed a multi-year, longitudinal research curriculum at our institution. This allowed for a comprehensive exploration of students’ perceptions across different stages of their medical training. The internal consistency of the survey domains and the robust statistical factor analysis conducted further support the validity of our findings. Furthermore, the thoughtful design, implementation, and delivery of our research curriculum, grounded in experiential learning theory [74] and scaffolded over multiple semesters, reflects best pedagogical practices in health profession education. The inclusion of allocated and protected hours, structured supervision, and outcome-focused assessments forms a model for embedding scholarship training within undergraduate medical education, which other institutions might want to adapt. A methodological consideration in this study is the modest sample size, which was constrained by the fact that the survey was distributed to the entire student population; increasing the sample was therefore not feasible. Traditional guidelines recommend subject-to-variable ratios of 5:1–10:1 for exploratory factor analysis [75,76], with 106 respondents for 31 items, our ratio of 3.4:1 falls below these conventional benchmarks. Nonetheless, psychometric indices (including KMO values, Cronbach’s alpha, and factor loadings) indicated strong internal consistency and suitability for factor analysis. Prior methodological studies have also demonstrated that smaller samples can yield acceptable factor solutions under certain conditions, particularly when communalities are high and loadings are strong [77,78]. On this basis, the current findings can be considered exploratory but still provide meaningful insights for curriculum evaluation.

Nevertheless, our study has limitations. The findings are based on a single institution and rely on cross-sectional, self-reported data, which limits the generalizability of our study [79] and may be subject to recall [80] and social desirability biases [81]. While our study provides valuable insight into student engagement and perceptions, it does not capture long-term behavioral or career outcomes, which would provide a more complete understanding of the curriculum’s impact. Moreover, with the absence of a baseline or control group, it is challenging to attribute outcomes solely to the longitudinal integrated research curriculum. Despite these limitations, the study offers valuable insights into students’ perceptions of a distinctive longitudinal, competency-based research module, supported by strong psychometric indices. As with all self-reported surveys, the possibility of careless responses cannot be excluded, although the consistency indices suggest reliability. Replication in larger cohorts or across institutions would strengthen the stability of these findings*.* These concerns are consistent with the broader challenges identified in medical education research, as reported by Cook and Artino [82], who stress the importance of considering psychological and contextual factors that influence learner behavior and self-reporting accuracy. In particular, the increasing emphasis on research engagement as a marker of medical residency competitiveness may consciously or unconsciously encourage inflated self-assessments of motivation, confidence, and productivity. This potential inflation, shaped by institutional culture and perceived expectations, highlights the need for cautious interpretation of self-reported outcomes in studies of this nature.

### 4.4. Implications for future research

To build on the current findings, future research should adopt longitudinal and mixed-methods study designs to examine how structured research training programs might influence medical students’ engagement, confidence, and scholarly output across a period of time. Integrating qualitative approaches, such as interviews or focus groups, could also provide a deeper understanding of how students internalize research experiences and overcome barriers to participation. Hart et al. [83] suggest that transformative learning models benefit from ongoing reflection and contextual relevance, which future studies could explore in diverse academic settings. Furthermore, examining faculty mentorship, institutional culture, and curriculum design using established frameworks such as those discussed by Benbassat [84], and Kaufman and Mann [85] can help identify scalable strategies for embedding research education within medical training. Broadly speaking, aligning future scientific inquiries with robust theoretical underpinnings will be critical in refining the role of research training in undergraduate medical education.

## 5. Conclusion

Our study affirms the value of structured research training, early exposure to research activities, and effective mentorship in fostering confidence, resilience, and positive attitudes toward research among undergraduate medical students. By embedding a mandatory longitudinal research module that actively engages students in the research process, the curriculum responds to calls within medical education to strengthen research literacy and evidence-based competencies. Further contributing to the limited regional evidence on integrated, compulsory research training. Importantly, students continued to engage in research activities during their subsequent clinical training, suggesting sustained research engagement beyond course completion. Together, these findings may inform ongoing local curriculum refinement and support the design of similar initiatives in other educational contexts.

## Supporting information

S1 File Raw data supporting the findings of this study. (XLSX)

S2 File Survey used in the study. (DOCX)
