# Supplementary material for: Exploring undergraduate medical students’ perception of an integrated longitudinal research curriculum within a competency-based framework
Source: PLoS One. 2026 Feb 23;21(2):e0343409. doi: 10.1371/journal.pone.0343409 (PMC12928465; doi:10.1371/journal.pone.0343409)
Supplement: S2 File — (DOCX) [file pone.0343409.s002.docx]

**Questionnaire**

1. Do you consent to participate in this study? Yes/no
2. What is your gender? Male/Female
3. How old are you (years)? *FREE TEXT*
4. What is your country of citizenship? *FREE TEXT*
5. What is your MBRU graduating class? 2022/2023/2024/2025/2026
6. Have you been awarded a degree from a university other than MBRU? Yes/No
7. How many articles (peer-reviewed) have you completed and published? If none type “0”. *FREE TEXT*
8. How many articles authored by you are under revision or preparation? If none type “0”. *FREE TEXT*
9. How many posters did you present from MBRU? If none type “0”. *FREE TEXT*
10. How many oral presentations have you delivered for MBRU that catered to research? If none type “0”. *FREE TEXT*
11. Have you won any research awards? If none type “0”, or type in the name of the award received. *FREE TEXT*
12. In your opinion at what point in your MD medical training do you think it is important to learn research methodology? FREE TEXT
13. Do you feel that completing the 5-integrated research courses during your first 3 years at MBRU helps you to become a better medical physician? If so, can you give an example. FREE TEXT
14. In your experience, do you believe that clinicians are receptive to research mentorship by you as a MBRU student? What factors do you think contribute to the clinician’s willingness to learn from you? FREE TEXT
15. **Please indicate your level of agreement with each of the following statements using the scale provided, from Strongly Agree to Strongly Disagree.**

|  | **Strongly Agree** | **Agree** | **Somewhat Agree** | **Neutral** | **Somewhat Disagree** | **Disagree** | **Strongly Disagree** |
| --- | --- | --- | --- | --- | --- | --- | --- |
| 1. I feel anxious and scared when designing or conducting research. I become nervous and scared when I must conduct or practice research |  |  |  |  |  |  |  |
| 1. I feel that students should receive training and information on designing and conducting research |  |  |  |  |  |  |  |
| 1. I did not have adequate training to initiate my research design |  |  |  |  |  |  |  |
| 1. I feel that conducting research is not necessary for obtaining academic or clinical recognition |  |  |  |  |  |  |  |
| 1. I face challenges when analysing research data, especially when it comes to statistical analysis |  |  |  |  |  |  |  |
| 1. Analysing research data causes me stress and anxiety |  |  |  |  |  |  |  |
| 1. I find it difficult to write a research manuscript |  |  |  |  |  |  |  |
| 1. Conducting research and publishing for career advancement are stressful |  |  |  |  |  |  |  |
| 1. I have difficulty understanding research design concepts |  |  |  |  |  |  |  |
| 1. When designing or conducting a research study, do you worry about making mistakes that could affect the validity of your findings? |  |  |  |  |  |  |  |
| 1. Research is not essential for me to fulfil my responsibilities in my current professional career |  |  |  |  |  |  |  |
| 1. Research will contribute to the advancement of my professional clinical career |  |  |  |  |  |  |  |
| 1. I am interested in conducting and developing my own research projects |  |  |  |  |  |  |  |
| 1. Research helps me understand how my MD courses relate to my clinical practice |  |  |  |  |  |  |  |
| 1. Conducting research is beneficial for most medical students |  |  |  |  |  |  |  |
| 1. Research can have a negative impact on my wellbeing by creating stress and anxiety |  |  |  |  |  |  |  |
| 1. Students should be informed about the clinical and translational significance of research |  |  |  |  |  |  |  |
| 1. I intend to apply principles of research outside of my professional practice |  |  |  |  |  |  |  |
| 1. The skills taught and acquired in the research courses will benefit my future clinical practice and career |  |  |  |  |  |  |  |
| 1. Research is essential to evolve into an evidence-based medical practitioner |  |  |  |  |  |  |  |
| 1. The learning objectives covered in the research courses have helped me develop my written and communication skills |  |  |  |  |  |  |  |
| 1. Research is not required outside of my professional practice |  |  |  |  |  |  |  |
| 1. Research training is an essential part of my professional clinical training |  |  |  |  |  |  |  |
| 1. Research is not a key component for me to perform my responsibilities in my current professional career |  |  |  |  |  |  |  |
| 1. Conducting research can be difficult most of the time |  |  |  |  |  |  |  |
| 1. I am interested in learning about the specific requirements to become a successful researcher |  |  |  |  |  |  |  |
| 1. I will empower my clinical practice with research |  |  |  |  |  |  |  |
| 1. Research-driven thinking is important outside of my professional practice |  |  |  |  |  |  |  |
| 1. Research is a complex and complicated activity |  |  |  |  |  |  |  |
| 1. Research is essential for becoming a safe and competent clinician |  |  |  |  |  |  |  |
| 1. I am willing to commit time to a research project including study design, data collection, analysis, and write-up |  |  |  |  |  |  |  |
